# Evolution of RNA viruses in trypanosomatids: new insights from the analysis of *Sauroleishmania*

**DOI:** 10.1007/s00436-023-07928-x

**Published:** 2023-07-25

**Authors:** Donnamae Klocek, Danyil Grybchuk, Lucie Tichá, Jan Votýpka, Petr Volf, Alexei Yu. Kostygov, Vyacheslav Yurchenko

**Affiliations:** 1 Life Science Research Centre, Faculty of Science, University of Ostrava, Ostrava, Czechia; 2 Central European Institute of Technology, Masaryk University, Brno, Czechia; 3 Department of Parasitology, Faculty of Science, Charles University, Prague, Czechia; 4 Institute of Parasitology, Biology Centre, Czech Academy of Sciences, České Budějovice, Czechia

**Keywords:** *Leishmania* (*Sauroleishmania*) *adleri*, *L.* (*S.*) *gymnodactyli*, *L.* (*S*.) *hoogstraali*, *L.* (*S*.) *tarentolae*, *Narnaviridae*, LRV2

## Abstract

RNA viruses play an important role in *Leishmania* biology and virulence. Their presence was documented in three (out of four) *Leishmania* subgenera. *Sauroleishmania* of reptiles remained the only underinvestigated group. In this work, we analyzed the viral occurrence in *Sauroleishmania* spp. and detected RNA viruses in three out of seven isolates under study. These viruses were of two families—*Narnaviridae* and *Totiviridae*. Phylogenetic inferences demonstrated that totiviruses from *L. adleri* and *L. tarentolae* group together within a larger cluster of LRV2s, while a narnavirus of *L. gymnodactyli* appeared as a phylogenetic relative of narnaviruses of *Blechomonas* spp. Taken together, our work not only expanded the range of trypanosomatids that can host RNA viruses but also shed new light on the evolution and potential routes of viral transmission in these flagellates*.*

**Supplementary Information:**

The online version contains supplementary material available at 10.1007/s00436-023-07928-x.

## Introduction

Leishmaniasis is a neglected parasitic disease threatening millions of people worldwide (WHO 2023). It is caused by flagellated protists of the genus *Leishmania*, which are mainly transmitted to vertebrate hosts by blood-feeding female phlebotomine sand flies (Diptera: Psychodidae) (Bruschi and Gradoni 2018). The genus comprises four subgenera, from which *Leishmania*, *Viannia*, and *Mundinia* are associated with human diseases, while members of the subgenus *Sauroleishmania* are restricted to reptiles (Espinosa et al. 2018; Kostygov et al. 2021b).

Although *Leishmania* in reptiles was first described over a century ago (Wenyon 1920), information about their life cycle is still limited, likely because they were not considered pathogenic to humans. Nevertheless, several historical and recent reports documented the presence of *Sauroleishmania* spp. in mammals, including dogs and humans (Adler 1962; Coughlan et al. 2017; Latrofa et al. 2021; Mendoza-Roldan et al. 2022b; Pombi et al. 2020).

*Sauroleishmania* has been isolated from a range of reptiles, mostly lizards and geckos of the families Agamidae, Gekkonidae, Lacertidae, Scincidae, and Varanidae (Belova 1971; Wilson and Southgate 1979), and sand flies of the genus *Sergentomyia*, which are considered the main vectors as they preferentially feed on cold-blooded vertebrates (Killick-Kendrick 1990). However, sand flies of the genus *Phlebotomus* are also susceptible to *Sauroleishmania* infection *in vitro* and *in vivo* (Tichá et al. 2021; Tichá et al. 2022).

It is now generally accepted that *Sauroleishmania* has evolved from the mammal-infecting parasites and all its 21 described species form a monophyletic group within the genus *Leishmania* (Akhoundi et al. 2016; Lukeš et al. 2018). The type species, *L.* (*S.*) *tarentolae*, has been extensively studied and is commonly used as a laboratory model in many fields, including biotechnology (Breitling et al. 2002; Klatt et al. 2019; Mendoza-Roldan et al. 2022a).

Numerous studies have reported presence of RNA viruses in representatives of the family Trypanosomatidae. To date, five different families of viruses were reported to infect these flagellates, of which *Leishbuviridae*, *Narnaviridae*, and *Totiviridae* are the most frequent (Grybchuk et al. 2018a; Grybchuk et al. 2018c).

Narnaviruses (naked RNA viruses) are capsid-less positive-strand RNA cytoplasmic elements encoding a single RNA-dependent RNA polymerase (RDRP) protein, although a multi-segmented narna-like virus has been described from the trypanosomatid *Leptomonas seymouri* (Kraeva et al. 2015; Lye et al. 2016). *Narnaviridae*, along with RNA bacteriophages (*Leviviridae*), mitochondrial capsidless RNA elements of eukaryotes (*Mitoviridae*), and plant viruses (*Botourmiaviridae*), belong to the phylum *Lenarviricota*. Based on phylogenetic inferences, it has been postulated that capsid-less mito- and narnaviruses diverged upon eukaryogenesis from an RNA phage that infected alphaproteobacteria, the ancestors of mitochondria (Koonin et al. 2015; Sadiq et al. 2022; Wolf et al. 2018). Narnaviruses of Trypanosomatidae are not monophyletic, and their evolution was likely shaped by several horizontal transfers (Grybchuk et al. 2018c).

*Leishmania* RNA virus (LRV) is a double-stranded RNA virus of the family *Totiviridae* infecting trypanosomatids from two genera: *Leishmania* (LRV1/2) (Scheffter et al. 1995; Stuart et al. 1992) and *Blechomonas* (LRV3/4) (Grybchuk et al. 2018c). These viruses form non-enveloped icosahedral virus particles about 40 nm in diameter (Procházková et al. 2021). No cellular receptors have been identified for LRVs. Thus, vertical inheritance is thought to be the predominant mode of viral transmission resulting in general co-evolution of LRVs and *Leishmania* spp. (Cantanhêde et al. 2021; Widmer and Dooley 1995). Notably, occasional horizontal transfers (both intra- and interspecific) have been also reported (Kostygov et al. 2021a). Such transfers are possible owing to the exploitation of host’s exosomes as vehicles for transmission between flagellates (Atayde et al. 2019; Lafleur and Olivier 2022; Olivier and Zamboni 2020). Infrequent mating events may also contribute to horizontal transmission of viruses in trypanosomatids (Akopyants et al. 2009; Rougeron et al. 2010; Sádlová et al. 2011).

There is strong evidence that LRVs provide a survival advantage to *Leishmania guyanensis* and *L*. *aethiopica* in vertebrate hosts through upregulation of pro-inflammatory cytokines facilitating the spread of parasites from the initial infection site (de Carvalho et al. 2019; Ives et al. 2011; Zangger et al. 2014). The LRV presence also downregulates apoptotic pathways and promotes parasite persistence (Eren et al. 2016). Of note, the molecular mechanisms governing viral maintenance may differ between LRV species (Saura et al. 2022).

In this work, we analyzed the viral occurrence in *Sauroleishmania* spp. and detected RNA viruses in three out of seven analyzed isolates. These viruses belong to two families—*Narnaviridae* and *Totiviridae*. Phylogenetic analyses showed totiviruses from *L. adleri* LV30 and *L. tarentolae* LV108 group together within a larger cluster of LRV2s, while a narnavirus of *L. gymnodactyli* LV247 appeared to be a phylogenetic relative of narnaviruses of *Blechomonas* spp. Taken together, our work expands the range of trypanosomatids that can host RNA viruses to include *Sauroleishmania*, the only *Leishmania* subgenus that has not been scrutinized in this respect so far.

## Materials and methods

### Isolates and cultivation

Seven cultures of *Leishmania* (*Sauroleishmania*) were examined: *L.* (*S*.) *adleri* RLIZ/KE/1954/1433 (LV30) isolated from the common long-tailed lizard *Latastia longicaudata* in Kenya in 1954 (Heisch 1958); *L.* (*S*.) *gymnodactyli* RGEC/SU/1964/Ag (LV247) isolated from the agamid lizard *Trapelus* (*Agama*) *sanguinolenta* in Turkmenistan in 1964 (Saf'janova 1966); *L.* (*S*.) *hoogstraali* RHEM/SD/1963/NG-26 (LV31) isolated from the Mediterranean house gecko *Hemidactylus turcicus* in Sudan in 1963 (McMillan 1965); *L.* (*S*.) *tarentolae* RTAR/IT/1981/ISS21-G6c (ISS21) isolated from the common wall gecko *Tarentola mauritanica* in Italy in 1981 (Pozio et al. 1986); *L.* (*S*.) *tarentolae* RCYR/IT/1981/ISS24-CK3 (ISS24) isolated from the Kotschy's gecko *Mediodactylus* (*Cyrtodactylus*) *kotschyi* in Italy in 1981 (Pozio et al. 1983); *L.* (*S*.) *tarentolae* IMIN/IT/2017/ISS3200RM-5 (ISS3200) isolated from the sand fly *Sergentomyia minuta* in Italy in 2017 (Di Muccio et al. 2015); and *L.* (*S*.) *tarentolae* RTAR/SE/67/G10 (LV108) isolated from the white-spotted wall gecko *Tarentola annularis* in Senegal in 1967 (Ranque 1973). This is one of the most comprehensive collections of the currently available *Sauroleishmania* isolates. Cells were cultivated as described previously (Tichá et al. 2022) and their identities were confirmed as in (Yurchenko et al. 2006).

### Screening for dsRNA and next-generation sequencing

Trypanosomatid cultures were screened as described previously (Grybchuk et al. 2020). In short, 50 μg of total RNA from each strain was digested with DNase I (Thermo Fisher Scientific, Carlsbad, USA) and S1 nuclease (Sigma-Aldrich, St. Louis, USA) and analyzed by gel electrophoresis. The three gel-positive samples were sequenced at Macrogen (Seoul, South Korea) following the protocol established before (Kleschenko et al. 2019).

### Sequence data processing and phylogenetic inferences

The raw sequence reads were trimmed with Trimmomatic v. 0.40 (Bolger et al. 2014), assembled *de novo* in Trinity v. 2.13.2 (Haas et al. 2013), and mapped back to the obtained contigs using Bowtie2 v. 2.4.4 (Langmead and Salzberg 2012) and SAMtools v. 1.17 (Danecek et al. 2021). BEDTools v. 2.30.0 software was used to estimate read coverage (Quinlan 2014). The identity of each contig was determined by running BLASTN (BLAST+ v. 2.13.0 (Camacho et al. 2009)) against a custom database of publicly available trypanosomatid genomes and BLASTX (DIAMOND v. 2.0.2 (Buchfink et al. 2021)) against UniClust50 protein database (Mirdita et al. 2017). All viral contigs were found by BLASTX. In addition, unmatched contigs were checked for the presence of long ORFs (as it usually the case for viral genomes); however, none was found.

The LRV phylogeny was inferred from concatenated protein sequence alignment of capsid and RDRP genes. The dataset was taken from previous work (Kostygov et al. 2021a). Each gene was aligned iteratively in MAFFT v. 7.490 (Katoh and Standley 2013) with G-INS-i algorithm and trimmed with a range of gap thresholds in TrimAl v. 1.4 (Capella-Gutiérrez et al. 2009). The optimal alignment length (665 positions at 0.7 gap threshold for capsid and 828 positions at 0.6 gap threshold for RDRP) and substitution model (LG + I + F + G4 for both capsid and RDRP) were selected based on the average bootstrap support value (ultra-fast bootstraps in IQ-TREE 2 v. 2.2.2.6 (Minh et al. 2020)). The respective alignments were concatenated and subject to maximum likelihood (ML) phylogenetic inference with 1,000 thorough bootstrap replicas in IQ-TREE 2 without partitioning. Bayesian tree was inferred in MrBayes v. 3.2.7. (Ronquist et al. 2012) with default settings and the same model as in the ML analysis. The tree was rooted at the midpoint.

The relationships of LRV2 viruses were inferred based on nucleotide sequences obtained here and all those available in the GenBank. The alignment was performed in MAFFT as above, but no trimming was applied. The ML analysis in IQ-TREE followed the same strategy, but the best automatically selected model was TIM2 + F + I + G4 and the branch support was estimated using 1,000 thorough bootstrap replicates as above.

The narnavirus phylogeny was inferred from the protein sequence of the RDRP gene. The dataset contained representatives of three families of *Lenarviricota*: *Mitoviridae*, *Narnaviridae*, and *Botourmiaviridae*. The alignment and trimming were done as above resulting in 496 positions long alignment obtained at 0.8 gap threshold and the best-fit model LG + I + F + G4. The ML and Bayesian phylogenies were inferred as above. The tree was rooted with *Mitoviridae* as an outgroup based on previous studies (Sadiq et al. 2022; Wolf et al. 2018).

## Results

### Three more species of *Leishmania* revealed to host RNA viruses

**Fig. 1:**
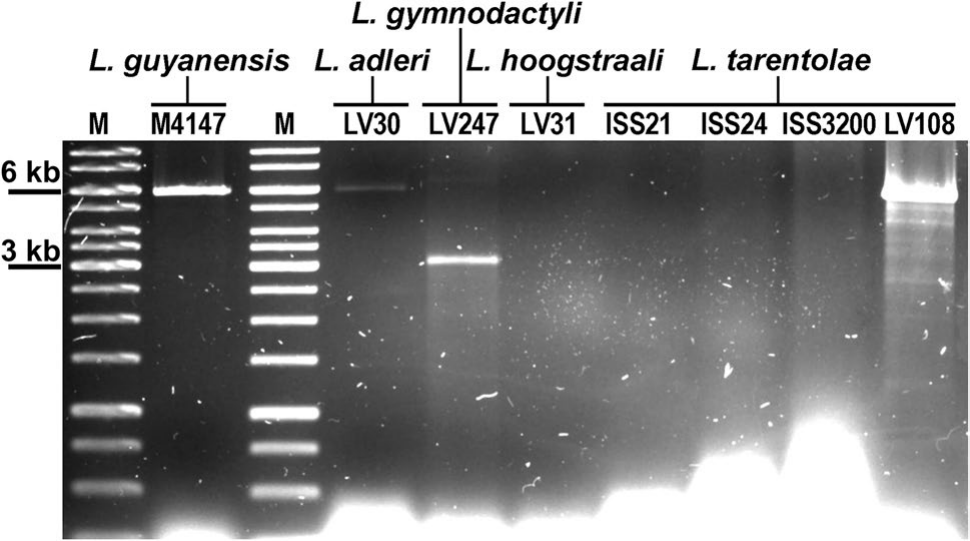
dsRNA agarose gel with screened *Sauroleishmania* isolates. *L*. *guyanensis* Lg-M4147 was used as a positive control. M - 1 kb DNA ladder

The gel analysis revealed that three out of seven isolates were positive for dsRNA (Fig. 1). *Leishmania* (*S*.) *adleri* LV30 displayed a single dsRNA band with similar mobility of approximately 6 kb, but at least by one order of magnitude lower intensity, as compared to that of the LRV1-4 from *L.* (*V*.) *guyanensis* M4147 that was used here as a positive control (Zakharova et al. 2022). On the contrary, a single dsRNA band of nearly the same size detected in *L*. (*S*.) *tarentolae* LV108 was severalfold brighter than that in the control. BLAST searches identified LRV2 in these two isolates of *Sauroleishmania*.

*Leishmania* (*S*.) *gymnodactyli* LV247 had a prominent major band of approximately 3 kb along with a fainter band that migrated at about 6 kb. The global BLASTX (all contigs versus clustered UniProt DB) detected only a narnavirus, which corresponded to the lower bright band, while the identity of the faint upper band could not be reliably established. One assembled contig of about 5.4 kb had no detectable ORFs and, as such, returned no BLASTX or BLASTN hits. Other contigs ranging between 5.5 and 7 kb had hits to the *Leishmania* genome. No dsRNA bands were documented in *L.* (*S*.) *hoogstraali* LV31 or three *L.* (*S*.) *tarentolae* isolates (ISS21, ISS24, and ISS3200).

### Phylogenetic position of the viruses from *Sauroleishmania*

**Fig. 2:**
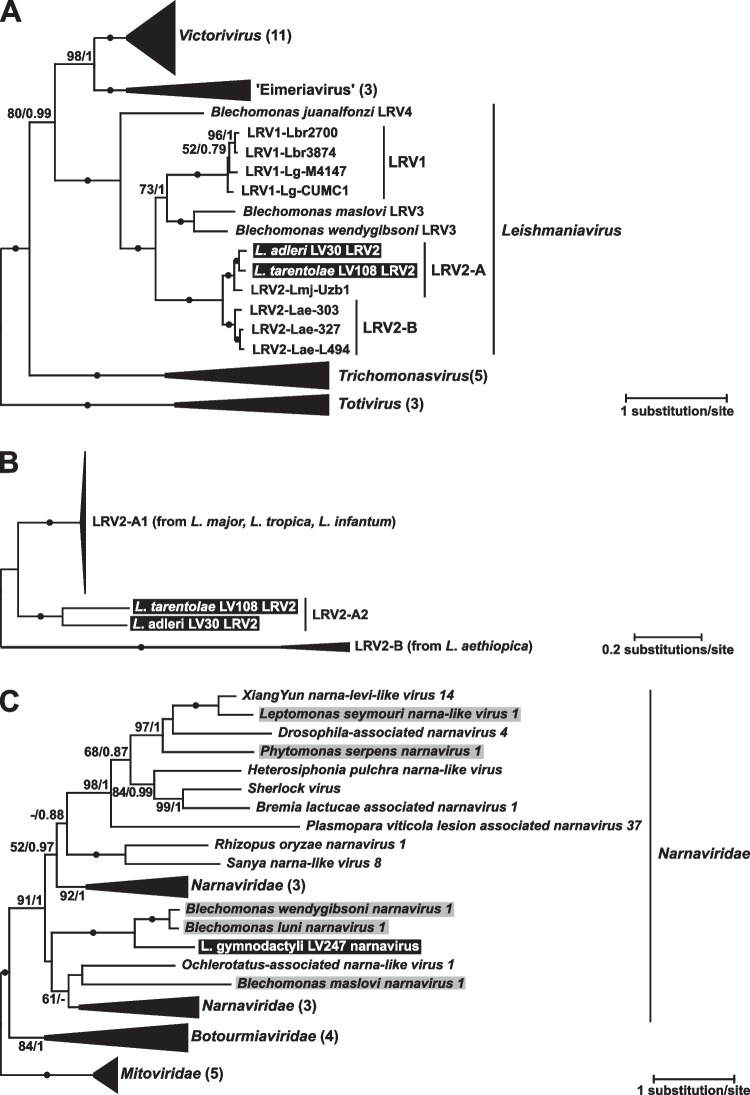
Phylogenetic position of viruses from *Sauroleishmania*. **A** Midpoint-rooted maximum likelihood tree based on concatenated capsid and RDRP amino acid sequences of selected *Totiviridae*. **B** Maximum likelihood tree of LRV2 based on nucleotide sequences of RDRP rooted with LRVs from *L. aethiopica*. **C** Maximum likelihood tree based on RDRP amino acid sequences of selected *Narnaviridae* and rooted with *Mitoviridae*. **A−B** Clades were collapsed for better visibility with a number of samples indicated in brackets. Viruses reported in this study are highlighted in black, narnaviruses previously found in other trypanosomatids are highlighted in gray. Numbers at branches represent bootstrap supports and Bayesian posterior probabilities; circles indicate absolute support (100/1); values below 50% or 0.5 are replaced with dashes or not shown

The phylogenetic analyses unambiguously (as judged by absolute statistical supports) demonstrated that the viruses from *L.* (*S.*) *adleri* and *L.* (*S.*) *tarentolae* LV108 belong to the LRV2, specifically to the clade associated with *L.* (*L.*) *major*. We denoted this clade as LRV2-A, as opposed to LRV2-B, containing viral sequences from *L.* (*L.*) *aethiopica* (Fig. 2A). To understand the relationships of the new viruses with their relatives from the LRV2-A clade, we performed an additional phylogenetic analysis using all nucleotide RDRP sequences of LRV2 available in the GenBank (Figs. 2B and S1). Although most of these sequences were rather short (ranging between 263 and 520 nt) preventing detailed resolution of their relationships (Fig. S1), it became obvious that the LRV2-A clade has a well-supported deep split between the viruses from *L.* (*Sauroleishmania*) [LRV2-A2] and those from *L.* (*Leishmania*) [LRV2-A1]. The latter subclade mostly contains viral sequences from *L.* (*L.*) *major* and only a few (nine sequences of 4 distinct haplotypes from *L.* (*L.*) *tropica* and one from *L.* (*L.*) *infantum*). We could not document host species-specific phylogroups in the LRV2-A1 clade.

The virus from *L.* (*S.*) *gymnodactyli* nested within the speciose family *Narnaviridae* with the closest relatives being the two sister viruses from trypanosomatids of the genus *Blechomonas*, namely, *Blechomonas wendygibsoni narnavirus 1* and *Blechomonas luni narnavirus 1* (Grybchuk et al. 2018c) (Fig. 2C). Interestingly, while statistical supports varied across the tree, the two internal branches determining such relationships between these three species showed maximal values for bootstrap percentage and posterior probability.

## Discussion

Our screening of *Sauroleishmania* isolates revealed viruses in three species. Out of these, the first two, namely, *L. adleri* and *L. tarentolae*, harbored the same viral species, which has been characterized earlier—*Leishmania RNA virus 2* (LRV2), while a new virus has been discovered in *L. gymnodactyli*. Although being new, it belongs to *Narnaviridae*, a group, the members of which has been repeatedly recorded in various trypanosomatids (Grybchuk et al. 2018a; Grybchuk et al. 2018c; Lye et al. 2016). Interestingly, only one *L. tarentolae* isolate (that from Senegal) harbored LRV2, while those three obtained in two different regions of Italy from three distinct animal species (one sand fly and two lizards) tested negative. This suggests that the distribution of viruses may be region-specific. However, it cannot be excluded that such a result is biased because of the small sample size.

Our findings shed a new light on evolution of viruses in trypanosomatids, which represents a quaint mixture of co-evolution and horizontal transfers. It was previously known that LRV2 is subdivided into two clades designated here as LRV2-A and LRV-2B for viruses from (mainly) *L. major* and *L. aethiopica*, respectively (Kostygov et al. 2021a). The divergence of these two groups is so ancient that the viruses belonging to them already acquired structural differences in their genome (Grybchuk et al. 2018b). In the current work, we revealed that the LRVs of *Sauroleishmania* fall into the LRV2-A clade, and this result can be explained by occasional co-infections of a common sand fly vector by parasites from these two subgenera (das Chagas et al. 2022; Latrofa et al. 2021; Mendoza-Roldan et al. 2022b; Pombi et al. 2020; Saf'janova 1991; Saf'janova et al. 1976). However, the additional phylogenetic analysis with all available LRV2 sequences suggests that such a viral transfer is not recent as judged by a relatively deep split between the LRV2-A1 of *L.* (*Leishmania*) and LRV2-A2 of *L.* (*Sauroleishmania*). Considering the limited number of analyzed isolates, we cannot judge whether this transition was unique. However, we argue that establishment of the infection in a phylogenetically distant and, therefore, physiologically different host is challenging and such events should be rare. Conversely, all documented transitions of LRV2-A1s from *L. major* to phylogenetically closer *L. tropica* and *L. infantum* appear quite recent (Hajjaran et al. 2016; Nalçacı et al. 2019; Saberi et al. 2020; Yurchenko et al. 2023) and occurred independently in different lineages of this viral clade, as we demonstrated here for the first time by combining all the available sequences of these viruses.

The new narnavirus discovered in *L. gymnodactyli* provides another example of an interesting link between *Leishmania* and *Blechomonas.* The sister relationship of this virus to those from *B. wendygibsoni* and *B. luni* is reminiscent of the situation with leishmania viruses: LRVs from these two trypanosomatid genera represent a monophyletic group, but it comprises more lineages (Grybchuk et al. 2018c). Previously, we hypothesized that all narnaviruses in *Blechomonas* spp. could be acquired independently from other trypanosomatids. However, the phylogenetic position of the virus from *L. gymnodactyli* suggests that, at least in this case, the initial acquisition was followed by a horizontal transfer to another trypanosomatid. As it was argued in our previous study, the direction of this transfer was from *Leishmania* to *Blechomonas*, since a contact between trypanosomatids of these two genera could occur only in the gut of a flea, the host of *Blechomonas* spp. (Grybchuk et al. 2018c). Out of the two variants possible for LRVs, i.e., transfer in the gut of either adult fleas or larvae, the second one appears more plausible. Indeed, the presence of *Sauroleishmania* (reptilian parasites) in the gut of adult fleas, which feed on mammalian blood, is unlikely. However, the larvae developing in the dens of mammals are scavengers that can consume bodies of dead insects, including sand flies, frequently appearing in such biotopes. Interestingly, this intergeneric transfer of viruses became possible because of the ability of some sand flies (e.g., *Sergentomyia* spp.) to feed on both reptiles and mammals (Polanská et al. 2020; Tichá et al. 2023). The repeated events of viral transition between *Blechomonas* and *Leishmania* (one for narnaviruses and at least two for leishmania viruses) suggest that these might also involve *Leishbuviridae* (LBVs), which have been documented in the flea-infecting *Blechomonas*. To date, only a single representative of this viral family (*Leishmania martiniquensis* LBV1) was found in *Leishmania* (Grybchuk et al. 2020).

In conclusion, detection of RNA viruses in *Sauroleishmania* not only closed the gap in our knowledge about viral presence in different groups of *Leishmania* but also shed light on the viral evolution in trypanosomatids*.* At the same time, our study raises new questions requiring screening of more samples and functional studies to reveal the significance of these viruses in *Sauroleishmania* biology.

## Supplementary information

ESM 1 Fig. S1. Maximum likelihood phylogenetic tree of LRV2 viruses (detailed version of the Fig. 2B). The crossed branch is shown at 25% of its length. In LRV2-A1 clade species other than *L. major* are labeled and highlighted in gray. All other designations are the same as in Fig. 2.

## Data Availability

All sequence data obtained in this work were submitted to GenBank with the following accession numbers: OR192165 (narnavirus of *L. gymnodactyli* LV247), OR192166 (LRV2 of *L. adleri* LV30), and OR192167 (LRV2 of *L. tarentolae* LV108).
