# Supplementary figures and images for: Evolution of RNA viruses in trypanosomatids: new insights from the analysis of Sauroleishmania
Source: Parasitol Res. 2023 Jul 25;122(10):2279–86. doi: 10.1007/s00436-023-07928-x (PMC10495512; doi:10.1007/s00436-023-07928-x)

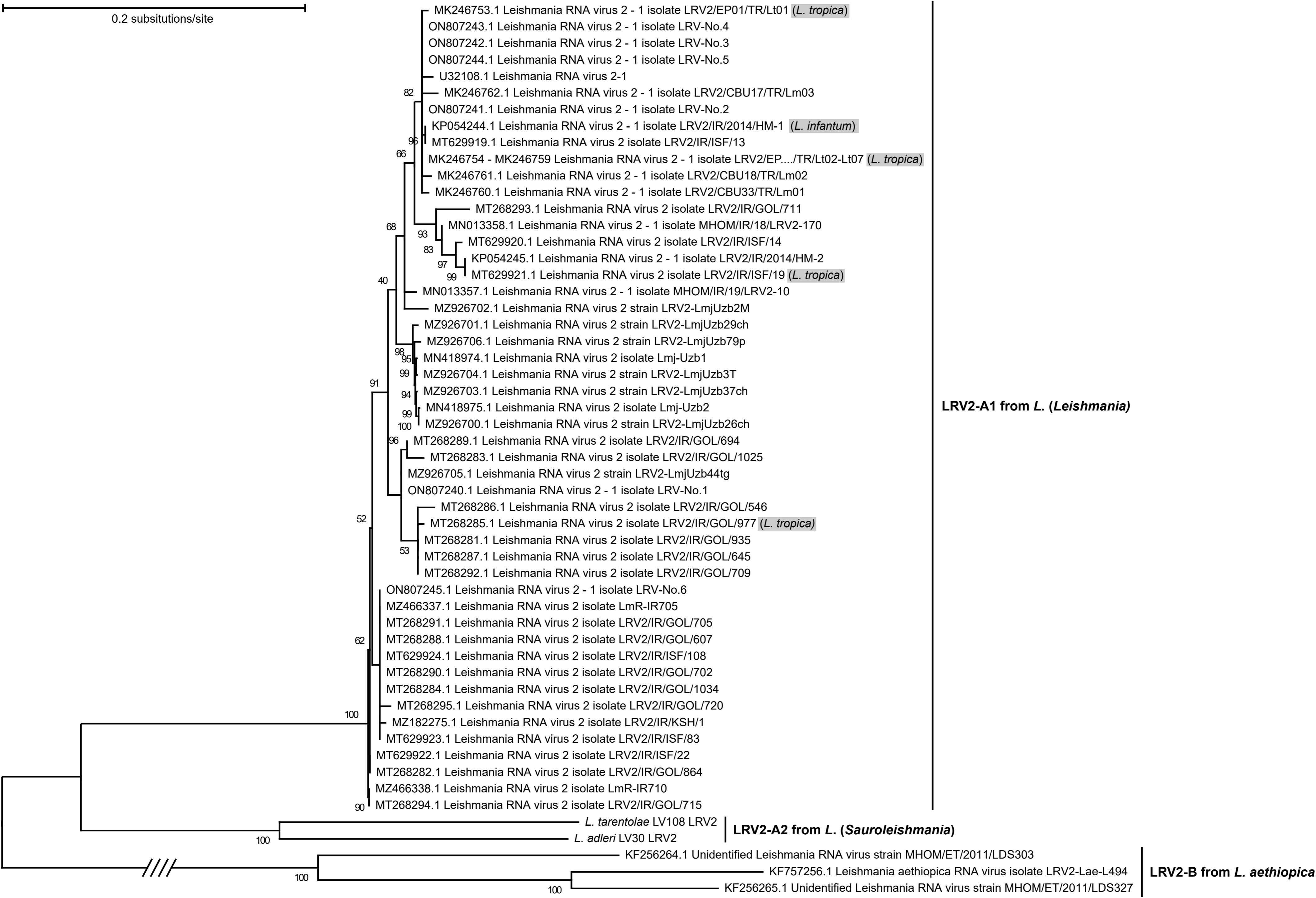

Supplement: Supplementary file 1 — Fig. S1. Maximum likelihood phylogenetic tree of LRV2 viruses (detailed version of the Fig. 2B). The crossed branch is shown at 25% of its length. In LRV2-A1 clade species other than L. major are labeled and highlighted in gray. All other designations are the same as in Fig. 2. [file 436_2023_7928_MOESM1_ESM.pdf]
